# Takotsubo syndrome in a Sardinian amyotrophic lateral sclerosis cohort

**DOI:** 10.1007/s00415-024-12681-x

**Published:** 2024-09-10

**Authors:** A. Maccabeo, M. I. Pateri, F. Pili, S. Pilotto, V. Pierri, A. Muroni, T. Ercoli, R. Montisci, M. F. Marchetti, A. Martis, L. Fazzini, G. Defazio, M. Puligheddu, G. Borghero

**Affiliations:** 1 Department of Medical Sciences and Public Health, Institute of Neurology, Policlinico Universitario di Monserrato, University of Cagliari, 09042 Cagliari, Italy; 2 Neurology Unit, AOU Cagliari, Hospital D. Casula Monserrato, Cagliari, Italy; 3 Department of Neurology, University of Sassari, Viale S. Pietro 10, 07100 Sassari, Italy; 4 Cardiology Unit, AOU Cagliari, Hospital D. Casula Monserrato, Cagliari, Italy; 5 Department of Medical Sciences and Public Health, Institute of Cardiology, University of Cagliari, Cagliari, Italy; 6 Department of Translational Biomedicine and Neurosciences, University of Bari “Aldo Moro”, Bari, Italy

**Keywords:** Takotsubo cardiomyopathy, ALS, Bulbar, Autonomic dysfunction

## Abstract

**Introduction:**

Amyotrophic lateral sclerosis (ALS) is known to be associated with varying degrees of autonomic and cardiovascular dysfunction. Recent case reports showed that ALS may be linked to Takotsubo syndrome (TTS). We assessed the frequency of TTS in an incident ALS cohort from Sardinia, Italy, and investigated the relationship of TTS with ALS course.

**Methods:**

We retrospectively examined a 10-year (2010–2019) incident cohort of ALS patients of Sardinian ancestry, reported TTS frequency and patients’ clinical characteristics. Following, we checked for TTS among patients with ALS onset after 2019 and focused on the same features as for the incident cohort.

**Results:**

Our incident cohort included 344 ALS patients and 5 of them (1.45%) developed TTS. All were female and their median onset age was 71.5 years (IQR 62.75–77). Two patients had spinal and three bulbar onset, though all patients had bulbar involvement and were at an advanced stage of disease (ALSFRS ≤ 25, King’s ≥ 3) at TTS diagnosis. We identified a potential TTS trigger in three patients (hospitalization for PEG placement, pneumonia). Among patients who had ALS onset after 2019, we identified a further TTS case and described it.

**Conclusion:**

TTS is not a rare condition in ALS. Female sex, bulbar involvement, and later age of disease onset may be important risk factors for developing this cardiac condition and a physical or psychological trigger is often observed. Despite autonomic dysfunction in ALS has been already demonstrated, the precise physiopathological mechanism underlying TTS needs to be further clarified.

## Introduction

Amyotrophic lateral sclerosis (ALS) is a fatal motor neuron disease characterized by progressive degeneration of spinal and bulbar upper and lower motor neurons [1]. Death generally occurs within 2–4 years from diagnosis due to respiratory failure. Increasing evidence is showing that ALS is a multisystem disease with significant variation in clinical presentation, including non-motor manifestations like behavioral, cognitive, metabolic, and autonomic alterations [2–5]. Cardiovascular autonomic dysfunction, consisting of decreased parasympathetic control and increased sympathetic activity, may appear since the early stages of the disease and is even more marked in patients with bulbar involvement [6, 7]. Chronic cardiac sympathetic hyperactivity is associated with sudden cardiac death and stress-induced cardiomyopathy in ALS patients [8]. Within this context, increased muscle sympathetic nerve activity (MSNA), a direct marker of sympathetic activity, was noticed in the early stages of ALS [9]. Takotsubo syndrome (TTS) is an acute heart failure syndrome triggered by stress-related adrenergic dysregulation, characterized by reversible left ventricular dysfunction and apical ballooning [10]. It is well-known that acute-onset or paroxysmal central nervous system (CNS) disorders may cause TTS, in particular stroke and epilepsy [11, 12]. Recently, a few reports raised the possibility of an association between TTS and ALS [10, 13–15], though the precise pathogenetic mechanism linking the two clinical entities has not been fully clarified. In this study, we assessed the frequency of TTS in an incident ALS cohort from Sardinia, Italy, and investigated the relationship of TTS with ALS course.

## Methods

We first checked for TTS in a recently described incident cohort [16] including 344 patients who developed ALS over a 10-year period (2010–2019). This cohort included ALS patients of Sardinian ancestry living in three administrative subdivisions (Cagliari, South Sardinia, and Oristano provinces) that covered about one-half of the whole island of Sardinia (Italy) and hosted nearly two-thirds of the whole Sardinian population. ALS diagnosis was made according to El Escorial revised criteria [17], and clinical phenotype was defined according to Chiò et al. [18]. Patients were classified by the ALS functional rating scale-revised (ALSFRS-R) score [19], and by King’s staging system [20]. DNA analysis was performed in a proportion of patients focused on SOD1, TARDBP, ATXN2, and C9ORF72 genes. TTS was diagnosed by our expert cardiologists according to the InterTAK Diagnostic Score [21]. We assessed TTS frequency in our incident cohort and focused on clinical features, genetic characteristics, and survival of ALS patients who presented this cardiomyopathy. Following, we retrospectively checked for TTS also from medical records of ALS patients seen at our center with disease onset after 2019 and we focused on the same features as for the incident cohort. The study was approved by the local Institutional Review Board and conducted in line with the ethical rules for data collection.

## Results

The incident cohort used in this study has been described in detail elsewhere [16]. In brief, the cohort included 344 patients (140 women and 204 men) whose mean onset age was 64.1 ± 11.2 years. Mean diagnostic delay was 14.7 ± 13.5 months, without significant differences between men and women. Classic limb onset phenotype was the most frequent clinical form (*n* = 168, 48.85%) followed by bulbar phenotype (*n* = 65, 18.19%), flail arm phenotype (*n* = 41, 11.9%), flail leg phenotype (*n* = 37, 10.8%), predominantly upper motor neuron ALS (*n* = 20, 5.8%), respiratory phenotype (*n* = 5, 1.4%) and predominantly lower motor neuron phenotype (*n* = 3, 0.9%).

In this incident cohort, we identified five TTS cases (1.45%) (Table 1, patients #1–#5). All were females and their mean onset age was 72 ± 5.4 years. TTS onset occurred 4–44 months after ALS onset. One patient (#2) had chronic ischemic heart disease and a positive ALS family history. Three patients (including the patient with positive family history) underwent testing for ALS-related genes (TARDBP, C9Orf72, ATXN2, and SOD1), but no relevant mutation was detected. Two patients had spinal and three bulbar onset, though all patients had bulbar involvement and were at an advanced stage of disease (ALSFRS-R score ≤ 25 and King’s score ≥ 3) at TTS diagnosis. One patient was hospitalized for PEG placement and other two had an ongoing pneumonia when TTS developed. One patient was mechanically ventilated at TTS diagnosis. All patients underwent a complete recovery of systolic function a few days after onset and one patient only (#2) had a recurrent TTS episode 1 year after the first. Survival time after TTS ranged from 1 to 25 months.

**Table 1:** Characteristics of case report (#6) and of other ALS patients who developed TTS (incident cohort 2010–2019)

Patient (sex)	#1 (F)	#2 (F)	#3 (F)	#4 (F)	#5 (F)	#6 (F)
ALS onset age (y)	63	74	77	69	77	62
Site of ALS onset	Left upper limb	Bulbar	Right lower limb	Lower limbs, bulbar	Bulbar	Bulbar
ALS genetics	Negative	Negative	NA	NA	NA	C9Orf72
ALS phenotype	Classic LO	BO	Classic LO	BO	BO	BO
TTS time (from ALS onset)	44 months	15 months	25 months	4 months	18 months	23 months
Symptoms at TTS	Chest pain	Excessive sweating	Dyspnea	NA	Chest pain, dyspnea	Chest pain, dyspnea
Bulbar at TTS	yes	yes	yes	yes	yes	yes
ALSFRS at TTS	17	21	25	12	14	16
King’s at TTS	4a	4b	3	4a	3	3
Ventilated at TTS	no	yes	no	no	no	no
FEVS at TTS	25%	NA	45%	NA	32%	25%
Cardiac follow−up	Complete recovery	2^nd^ TTS episode 1 year later	Complete recovery	NA	Complete recovery	Complete recovery
Cardiovascular comorbidities	A	IHD, HT	A	HT	HT	A
Intervening conditions	PEG placement 2 days earlier	A	Pneumonia	Pneumonia	A	Pneumonia
TTS–death (time)	25 months	23 months	1 month	5 months	1 month	−
ALS disease duration	69 months (†)	38 months (†)	26 months (†)	33 months (†)	18 months (†)	−

*BO* bulbar onset, *LO* limb onset, *IHD* ischemic heart disease, *HT* hypertension, *NA* data not available, *A* absent

Among patients who had ALS onset after 2019, we identified a further TTS case. This was a 64-year-old woman (#6) who started complaining of dysarthria, sporadic dysphagia, and weight loss around December 2021 (62 years old). After a complete neurological assessment, she received a diagnosis of clinically defined ALS 5 months later. She referred family history of ALS (father) and C9Orf72 expansion in heterozygosis was detected. Within the following months, dysarthria and dysphagia worsened and the patient developed neck extensor weakness with a dropped head. During summer 2023, she started complaining of weakness in four limbs prevalent at distal segments of upper limbs and reported multiple falls. In October 2023, the patient presented productive cough, fever, and, a few days later, dyspnea and chest pain. She was brought to the ER, where ECG showed sinus tachycardia (122 bpm), Q waves in V3-V6, ST-elevation in V3 with negative T waves in V4-V5. A blood test revealed elevation of troponin T (up to 2700 ng/L) and echocardiography revealed medial-apical akinesia with severe reduction of ejection fraction (25%). She was thus admitted to the Coronary Intensive Care Unit with suspicion of TTS. Her ALSFRS-R score at that time was 16 and her King’s score was 3. Due to a respiratory worsening, she underwent a tracheostomy 2 days after the hospitalization and PEG placement 3 weeks later. A cardiologic re-evaluation 1 month after the admission showed a complete remission of medial-apical akinesia and normalization of ejection fraction (60%), leading to the final diagnosis of TTS. She was then discharged from the hospital in a stable cardiac condition.

## Discussion

In our cohort, 1.45% of incident patients (5/344) developed TTS over ALS course. This finding is apparently consistent with the frequency estimates provided by prior reports [10, 11, 13]. It is worth noting, however, that prior studies identified cases from medical records of ALS patients seen at specialized clinics over a specific period; by contrast, our estimate relied on an incident cohort and this strengthens the accuracy of our findings. In fact, incident cohorts tend to better reflect ALS clinical phenomenology while prevalent cohorts tend to be younger, live longer, have a higher proportion of male patients and fewer with bulbar phenotype [19], all factors that may affect the possibility of TTS development. Interestingly, Choi et al. diagnosed TTS in nine out of 64 ALS patients (14.1%) who underwent transthoracic echocardiogram for acute dyspnea, chest pain, or preoperative cardiovascular workup. These findings suggest that TTS incidence could be underestimated due to a lack of awareness of this clinical entity. [10].

In our cohort, bulbar involvement and an advanced stage of the disease (King’s score ≥ 3) were the main factors timely associated with TTS development in all six subjects, regardless of disease duration and site of ALS onset. Bulbar neuropathological alterations have been repeatedly observed in ALS and have been linked to spread of the disease through contiguous anatomical structures rather than trans-synaptic propagation [13–22]. The involvement of autonomic bulbar nuclei determines denervation of autonomic cardiac nerves. Sympathetic hyperactivity secondary to autonomic impairment may lead to multi-vessel coronary spasm, myocardial stunning, and excessive transient ventricular afterload [7, 11, 15], and is a potential explanation of the high incidence of TTS in ALS [11]. A physical or psychological precipitating factor, such as invasive ventilation via tracheostomy, acute infections, or interventional endoscopy, causes an increase in circulating catecholamines that may act as a “second hit” and affect cardiac function [11]. Indeed, TTS is known to be preceded by a trigger factor in 70% of patients. In line with that, most of our patients developed TTS concurrently with hospitalization for PEG placement or infections.

Interestingly, all patients who developed TTS were women, and their mean onset age was 72 years. It is well-known that TTS incidence is higher in females among general population and approximately 90% of patients with TTS patients are women [23, 24]. However, previous studies on ALS patients developing TTS showed a very slight sex difference. Although random variability or other bias cannot be excluded, the high incidence of TTS in our female patients may be consistent with the observation that ALS peaks later in women and that women are more likely to have a bulbar onset typically associated with advanced age [25]. At the same time, postmenopausal estrogen deprivation may play a facilitating role in the pathogenesis of TTS [15, 24].

This study has strength and limitations. The incident population was similar to the general ALS population for most demographic and clinical features, including men preponderance, age at onset, and frequency of clinical phenotypes. This strengthens the accuracy of our findings. The difficulty in reporting symptoms, as for patients with cognitive impairment or artificially ventilated, the incorrect interpretation of symptoms and the possibility of sudden death from unknown cause are all conditions that may have led to underestimation of TTS frequency. Despite the foregoing limitations, the frequency estimate found in our incident ALS cohort suggests that TTS is not uncommon in ALS. Female patients, older than 65 years, with signs of bulbar involvement, at an advanced stage of the disease and in conjunction with precipitating factors have the greatest risk of developing TTS. Considering the potential reversibility of this condition if treated on time, it is highly recommendable to suspect TTS and perform a complete cardiovascular assessment in ALS patients who show signs or symptoms of cardiac dysfunction, acute exacerbation of dyspnea, and chest discomfort.

## Data Availability

Data supporting the findings of this study are available from the corresponding author (A.M.) on request.
